# Brief interventions for alcohol use disorders in low- and middle-income countries: barriers and potential solutions

**DOI:** 10.1186/s13033-022-00548-5

**Published:** 2022-08-08

**Authors:** Abhijit Nadkarni, Urvita Bhatia, Andre Bedendo, Tassiane Cristine Santos de Paula, Joanna Gonçalves de Andrade Tostes, Lidia Segura-Garcia, Marcela Tiburcio, Sven Andréasson

**Affiliations:** 1 Centre for Global Mental Health (CGMH), Department of Population Health, London School of Hygiene & Tropical Medicine, London, UK; 2 Addictions Research Group, Sangath, Porvorim, Goa India; 3 Department of Psychology, Health and Professional Development at Oxford Brookes University, Oxford, UK; 4 Department of Psychobiology, Universidade Federal de São Paulo (UNIFESP), Sao Paulo, Brazil; 5 Department of Health Sciences, Faculty of Sciences, University of York, York, UK; 6 Center for Research, Intervention and Evaluation on Alcohol & Drugs (CREPEIA), Department of Psychology, Universidade Federal de Juiz de Fora (UFJF), Juiz de Fora, Brazil; 7 Program on Substance Abuse, Public Health Agency of Catalonia, Barcelona, Spain; 8 Clincal Psychology and Health Department, Autonomous University of Barcelona, Barcelona, Spain; 9 Department of Social Sciences in Health, Instituto Nacional de Psiquiatría Ramón de la Fuente Muñiz, Mexico City, Mexico; 10 Department of Global Public Health, Karolinska Institutet, Stockholm, Sweden

**Keywords:** Brief interventions, Alcohol use disorders, Low- and middle- income countries, India, Latin America

## Abstract

Global alcohol consumption and harmful use of alcohol is projected to increase in the coming decades, and most of the increase will occur in low- and middle-income countries (LMICs); which calls for cost-effective measures to reduce alcohol exposure in these countries. One such evidence based measure is screening and brief intervention (BI) for alcohol problems. Some of the characteristics of BI make them a particularly appealing choice of interventions in low-resource settings. However, despite evidence of effectiveness, implementation of BI in LMICs is rare. In this paper we discuss barriers to implementation of BI in LMICs, with examples from Latin America and India. Key barriers to implementation of BI in LMICs are the lack of financial and structural resources. Specialized services for alcohol use disorders are limited or non-existent. Hence primary care is often the only possible alternative to implement BI. However, health professionals in such settings generally lack training to deal with these disorders. In our review of BI research in these countries, we find some promising results, primarily in countries from Latin America, but so far there is limited research on effectiveness. Appropriate evaluation of efficacy and effectiveness of BI is undermined by lack of generalisability and methodological limitations. No systematic and scientific efforts to explore the implementation and evaluation of BI in primary and community platforms of care have been published in India. Innovative strategies need to be deployed to overcome supply side barriers related to specialist manpower shortages in LMICs. There is a growing evidence on the effectiveness of non-specialist health workers, including lay counsellors, in delivering frontline psychological interventions for a range of disorders including alcohol use disorders in LMICs. This paper is intended to stimulate discussion among researchers, practitioners and policy-makers in LMICs because increasing access to evidence based care for alcohol use disorders in LMICs would need a concerted effort from all these stakeholders.

## Introduction

Alcohol and drug use is the fifth-leading risk factor for poor health in men globally, associated with 6.6% (2% in women) of disease [1]. Alcohol use is ranked as the seventh leading risk factor for premature death and disability [2]. Alcohol use disorders (AUDs) (chronic relapsing conditions characterized by an impaired ability to stop or control alcohol use despite adverse health, social, or occupational consequences) are the largest contributors to premature mortality amongst all mental and substance use disorders and account for 9.6% of DALYs and 44.4% of Years of Life Lost caused by these disorders [3]. Overall, alcohol use is associated with far more health loss for males than for females, with the attributable burden for men around three times higher than that for women [2]. However, there is growing evidence to indicate the increasing prevalence of alcohol consumption amongst women, and also other demographic groups such as adolescents and the elderly, including in low- and middle-income countries (LMICs) [4–12].

Between 1990 and 2017, global adult per-capita alcohol consumption increased from 5.9 to 6.5 L, and is expected to reach 7.6 L by 2030 [13]. Importantly, the annual growth rate of volume consumption per person (1997–2009) was 2.8% in LMICs compared to 1.1% in high-income countries (HICs) [14]. In such a scenario, global goals for reducing the harmful use of alcohol, as expressed by the WHO global alcohol strategy to reduce the harmful effects of alcohol, are unlikely to be achieved [13]. Given these developments, it becomes critical to ensure that cost-effective intervention measures are implemented to reduce alcohol exposure.

One such evidence based measure is brief intervention (BI) for alcohol problems, and some of the characteristics of BIs make them a particularly appealing choice of interventions in low-resource settings (particularly for hazardous and harmful drinking). BIs are a variety of structured techniques that aim to motivate behaviour change and are utilised to change risky alcohol use [15]. BIs can be adapted to different behaviours, settings and practitioners’ needs and hence can involve a variety of approaches. Table 1 summarises the essential characteristics of BIs in terms of types of BIs, key content, potential target groups, and settings in which they can be delivered.

**Table 1:** Essential characteristics of BIs

Approach	Brief (range: between 5 and 40 min), flexible, can be adapted to different behaviour contexts, settings, practitioners
Target group	Hazardous and harmful drinkers
Measures	Use of validated screening tools to identify drinking patterns
Content	Structured techniques focussed on motivating behaviour change including: Feedback on the person’s alcohol use and any alcohol-related harm Clarification as to what constitutes low-risk alcohol consumption; information on the harms associated with risky alcohol use; and benefits of reducing alcohol intake Advice on how to reduce alcohol intake Motivational enhancement Analysis of high-risk situations for drinking and coping strategies Development of a personalised plan to reduce alcohol consumption Referral to further treatments where appropriate
Types of BIs	Brief structured advice Motivational interviewing based BIs Digital BIs (e.g. app-based)
Settings in which BIs may be delivered	Primary care Specialist care Emergency care

In general, BIs are grounded in social-cognitive theory and incorporate some or all of the following elements: feedback on the person’s alcohol use and any alcohol-related harm; clarification as to what constitutes low-risk alcohol consumption; information on the harms associated with risky alcohol use; benefits of reducing alcohol intake; advice on how to reduce alcohol intake; motivational enhancement; analysis of high-risk situations for drinking and coping strategies; and the development of a personalised plan to reduce alcohol consumption [16]. Although there are several forms of BIs, the more commonly used approaches are brief structured advice and motivational interviewing (MI)-based interventions, and also interventions delivered electronically, through mobile phone applications and websites, where advice may be accessed more conveniently by patients [17].

The settings, target groups, and delivery agents described in studies of BIs vary [16]. When applied in primary care and emergency care they are generally delivered by general practitioners, generalist healthcare workers, nurse practitioners or psychologists. The target groups are hazardous and harmful drinkers who are not specifically seeking help for alcohol problems and the aim is reduced consumption and alcohol-related harms.

Brief structured advice lasts 5 to 10 min and is used when the time is limited and is relatively directive in nature e.g. providing feedback with information on drinking risk levels, and encouraging the reduction of consumption by simple advice, often accompanied by educational materials [18, 19]. It aims to evoke change in drinking behaviour, by providing individuals with information about their drinking and how they may reduce their consumption to sensible drinking levels [20]. On the other hand, MI-based interventions are more flexible and take a client-centred approach that avoids explicitly directive advice. Such interventions may last between 20 and 40 min and often include follow‐up sessions. Based on the principles of MI [21], it has been applied in contexts where there is more time and where trained professionals are available. BIs are usually delivered opportunistically for individuals who are not particularly seeking help for their alcohol use but whose use is a concern for the practitioner [22]. They are conceived as public health interventions, with the aim to detect alcohol use problems in the early stages and provide advice to reduce adverse consequences at individual and societal levels.

BIs for alcohol use disorders (AUDs) have been extensively researched over the last four decades [20, 23, 24], with a large body of evidence indicating their efficacy in primary care and emergency care. However, more recently, questions have been raised about the effectiveness of BIs [25, 26], especially the translation of the research evidence into implementation in routine practice, including the referral of more severe alcohol use problems from primary care to specialised addiction treatment (e.g. referrals are likely not to be successful because most patients are reluctant to seek specialized treatment for alcohol problems, and, when referred, are lost along the way) [27].

The current debate regarding the effectiveness of BIs is centred around the primary challenge of implementation at scale. Based on the efficacy studies of BIs, one can consider them as a highly competitive option for public health interventions to reduce alcohol related harm. Along with a range of other public health policies designed to reduce alcohol related harm, such as increased taxation of alcohol, regulation of alcohol advertising, and control of opening hours for alcohol retail outlets, BIs stand out as another effective policy measure, achieving large effects as measured by disability adjusted life years (DALYs) [28]. This conclusion however is based on the efficacy of BIs, and not taking implementation barriers into account. Despite their efficacy, if BIs cannot be implemented in practice, other evidence-based policies should be considered. It is with this perspective that the analysis of barriers to implementation of BIs becomes crucial.

Thus, although a large number of trials of BIs have been conducted, with modest outcomes in HICs, implementation in clinical practice remains elusive, calling for a rethinking of the core principles underlying these interventions. Furthermore, there is very limited evidence for both efficacy and effectiveness of BIs in LMICs, and implementation of BIs in LMICs is rare as well [29–31]. In summary, early identification and care for people with AUDs has received much less attention in LMICs than in HICs [31–33]. While lessons from HICs are important, implementation of BIs in LMICs involves a different set of contextual challenges [31, 34], and such possible barriers to BIs in LMICs, and solutions to overcome them, need further investigation.

## Methodology

We conducted a literature search through Medline, Google Scholar and SciELO to find and select published research covering a broad range of aspects on BIs in LMICs. Our inclusion criteria were (1) BIs for alcohol and drugs, (2) any kind of use ranging from risky/hazardous to substance use disorder, (3) any publication date, and (4) in LMIC settings. Our eligibility criteria were intentionally broad as the literature on BIs in LMICs is sparse and our primary objective was to provide the reader with the landscape of evidence base related to BIs in resource-constrained settings. Many of the identified studies from Latin America did not evaluate effects of BIs, and/or did not discuss implementation barriers. Therefore, we also included additional relevant publications in order to improve the description of the implementation strategies used in Latin America. Table 3 summarises evidence from studies reporting BI implementation experiences (barriers or effects).

We did not apply any date and language restrictions to the studies. JT summarized literature on barriers to implementation in LMICs. AB synthesised literature from Latin America and UB synthesised literature from India. Using a narrative synthesis approach [35], we summarize key examples below to highlight salient characteristics of the content and delivery of BIs in LMICs, with a focus on evidence related to the Latin American and Indian context as case studies representative of LMICs.

## Results

### Summary of barriers to alcohol BI implementation in LMICs

There are several barriers to implementation of BIs, the key ones being lack of financial and structural resources. In this paper, we will discuss these barriers, reflecting on specific examples from diverse low-resource settings. We specifically discuss two types of barriers seen in addictions care: demand (related to the felt need for services) and supply (related to the provision of services) side barriers. Table 2 provides a brief taxonomy of barriers, summarizing the evidence below into two major categories, demand side and supply side barriers.

**Table 2:** Barriers to BI implementation

Demand side	Supply side
Stigma associated with alcohol use disorders [29, 40–42]	Quality of BI implemented [24]
Lack of knowledge about available treatments [38]	Primary health care providers are not trained, are overburdened with existing responsibilities [31, 32, 34, 36, 37]
Low help-seeking rates for alcohol use disorders [38, 39]	Poor structural resources, including training and systems [34, 38]
Poor sensitisation among primary health care providers [36, 37]	Lack of financial resources and investment in BIs [31, 32, 38]
	Poor policy planning [48, 49, 66]

Although there are not many such studies from LMICs, there is no reason to believe that barriers to BI identified in HICs (e.g. lack of trained specialists) would not apply to LMICs. Studies from Latin America (and other LMICs such as South Africa) observed that factors influencing the implementation of BIs in routine primary care practice included the percentage of nurses trained in BIs, support visits, clinical workload, competing priorities, team work, early adoption, compatibility beliefs, perceived complexity of innovation, trialability and observability of BIs, social norms relative to substance use and the relationship between technicians and health professionals [31, 32, 36, 37].

Specifically, in LMICs, specialized services are limited or non-existent [32] and hence primary care is often the only possible alternative to implement BIs. However, health professionals in such settings generally lack training to deal with AUDs [36, 37], and often the level of engagement is slow [38]. This can lead to reluctance to address the problem due to concerns about patients’ reactions [37]. A great number of patients experiencing alcohol problems avoid disclosing such problems with healthcare providers and they usually seek treatment for other conditions that may be related to their alcohol use. This is one of the reasons why screening and appropriate management is crucial to reach this target population [38].

However, for healthcare professionals this is difficult within the context of intensive work hours, multiple jobs, low wages, poor working conditions, work overload, and reduced staff numbers in these settings [34]. One key obstacle that health professionals face is that patients have low motivation to participate in interventions for their drinking problems as they prefer to handle these problems by themselves [38]. Concerns about patient confidentiality and privacy, and lack of patient cooperation are other challenges that professionals describe [39]. These barriers to access add on to the existing large health disparities in LMICs where low socioeconomic position increases the severity of alcohol problems, which are then further aggravated by a lack of access to healthcare and other relevant services [31, 38].

Another barrier is the stigma related to AUDs, leading to limited acceptability and adherence to treatment [29, 40, 41]. As in HICs, stigma remains one of the greatest hindrances for substance users to access health services in LMICs. Additionally, in resource-poor settings such as LMICs, where the investment in health is already inadequate, interventions to reduce stigma and minimize its impact on users and family members are even less prioritized. Therefore, the same cultural and structural factors that enhance stigma also threaten development and maintenance of interventions aimed to deal with this problem [42].

Finally, there is limited willingness among policymakers to invest in implementation of BIs in LMICs. Some of this is a consequence of the mixed evidence about the effectiveness of BI- some encouraging [30, 43], other less so [24]. Furthermore, despite evidence from HICs, little is known about the effectiveness and feasibility of BIs in LMICs [37]. It is critical to develop the evidence base in LMICs where there are marked socioeconomic inequalities, and hence it is important to understand which types of BIs are appropriate for which target population and for which contexts. However, although one might acknowledge that such research is necessary, the resources for conducting such research in LMICs are limited [32].

### Strategies and new approaches for BIs: perspectives from selected LMICs

#### Latin America

Alcohol consumption represents a major health problem for Latin America. Countries with the most per capita consumption are Argentina, Brazil, Chile, Mexico, Panama, Paraguay, and Uruguay [44]. Several inequities present in Latin America such as living conditions and limited access to the health care system reinforce the impact of alcohol consumption and the related problems. Among key concerns are a higher mortality rate from alcohol‐attributable causes, emerging high-risk groups such as youth and women, and the consumption of high alcohol content beverages [45]. Although public health policies have a fundamental role in the reduction of alcohol-related mortality, there is no consensus among Latin American countries regarding current legislation on alcohol and public health.

BIs were first used in Latin America in the late 1980s in Brazil [46] and, since then, substantial efforts have been made to study them in various countries in Latin America. However, evidence from Cochrane reviews highlights a clear need for more evaluative research on BIs in LMICs [16]. Most studies of BIs in Latin America have been conducted in primary care settings, with small samples, and focused on process evaluation rather than efficacy or effectiveness, a major limitation that undermines the knowledge on the use of BI in this context.

Human resources in mental health in Latin America are scarce [47, 48], and there are high levels of professional turnover [49]. To some extent, this lack of human resources is also related to a well-documented lack of training [48, 50, 51]. Thus, several efforts in Latin America are aimed at training professionals in delivering BIs.

In Brazil, the countrywide distance-learning course SUPERA (https://www.supera.org.br) has offered more than 135,000 training opportunities in screening and BI, since 2006. Most professionals who completed the course applied BI in their work, and patients receiving the intervention had significantly reduced scores on the Alcohol, Smoking and Substance Involvement Screening Test (ASSIST) [52]. Additionally, the Regional Referral Centers on Drugs have also trained hundreds of health, social and justice professionals around the country [53]. Implementation studies from Brazil show that BI training helped to overcome the stigma and moralistic views about alcohol among professionals. Although neither of the studies evaluated effectiveness or efficacy of BIs, the authors highlighted the lack of integration within and between services and absence of support from policymakers or health administrators as relevant barriers for implementation of BIs [48, 49]. In Mexico, there have been advances in training professionals, prevention, and treatment, with over 400 centers offering BI-based services across the country; however, the impact of this program has not been fully evaluated [27]. The government’s initiative for implementing BI in Chile has helped multidisciplinary teams to carry out approximately 600,000 screenings and BI annually [53]. However, one of the largest pragmatic randomized controlled trial of BIs in Latin America conducted in a range of Chilean settings (health centers, emergency rooms and police stations) showed no evidence of the effectiveness of BI [41]. Finally, the use of web-based BI delivery in LA countries might help to overcome some implementation barriers, such as the lack of training, human resources, and financial support. In a World Health Organization (WHO) multicenter randomized controlled trial testing the efficacy of a web-based BI [54], preliminary findings from Brazil showed reductions of alcohol consumption [55].

Several studies considered the use of BI among vulnerable populations in LA. Studies from Mexico, Argentina, Brazil, and Colombia evaluated the use of BI among adolescent populations [56–62], but evidence is mixed and limited in terms of representativeness of the samples. For instance, the few randomized controlled trials [60, 62] vary with regard to the number of BI sessions (from one to four) and sample sizes (ranging from 25 to 58). A qualitative study assessing the feasibility of using BI for HIV/SIDA populations in Peru concluded that there are multiple barriers to the implementation of BIs through existing healthcare systems, including lack of awareness of substance use problems, lack of time and space, and shortage of specialized centers to refer patients [63]. These studies highlight the need for randomized controlled trials to examine effectiveness as well as scaling up of BIs.

Thus, relevant knowledge from Latin America is predominantly derived from experiences with BI training programs and some governmental implementation of BI as healthcare policy. These studies highlighted some barriers to BI implementation in Latin America. These include lack of engagement of policymakers and health service administrators [31], stigma among professionals [49, 64], scarcity of human resources [47], high levels of professional turnover [49], and lack of training [48, 49]. Finally, the majority of these studies from Latin America are from around 7 out of 33 nations; each one having its own political, sociocultural, economic, and epidemiological idiosyncrasies that should be considered while implementing BIs.

To summarize, the current evidence of BIs in Latin America is promising, but the lack of effectiveness studies remains a major limitation. Appropriate evaluation of BI efficacy and effectiveness in Latin America is undermined by lack of generalizability, and methodological limitations; and implementation is hampered by multiple barriers.

#### India

Alcohol is the second most common substance used in India, after tobacco [65]. Rapid socio-economic changes in India over the past couple of decades, have resulted in an increase in alcohol availability and consumption. Overall, although India has a relatively high abstinence rate, many people who do drink, do so problematically [66]. Despite this growing public health problem, the official policy response in India remains primarily focused on treatment services for AUDs in specialist settings, and within that, on the more severe problems of alcohol dependence [66].

Primary care in India offers opportunities for the early detection and management of AUDs. However, with addictions treatment being primarily situated in tertiary care, there is no routine screening conducted at primary care or in the community; resulting in a large treatment gap. Furthermore, there have been no systematic and scientific efforts to explore the implementation and evaluation of BIs in primary and community platforms of care in India.

In one of the first randomized controlled trials of brief motivational interviewing in any LMIC, a brief treatment delivered by lay counsellors to harmful drinkers in India led to positive and sustained effects on remission and abstinence, and was also demonstrated to be cost-saving from a health systems’ perspective [67, 68]. There is promise in the road ahead for AUD care in India, with emerging evidence on the use of innovative solutions which can potentially revolutionize how treatment of addictions is conceptualized [69]. Chand and colleagues have developed a system for capacity building in the practice of addictions treatment, by linking non-specialist health professionals with specialists for remote supervision [70]. In recent times, India’s health system has undergone a paradigm shift in health care delivery. The recently launched “Ayushman Bharat” (Healthy India) [71] national level initiative (which has envisioned universal health coverage) covers preventative and therapeutic interventions for non-communicable diseases at the community level, through Health and Wellness Centres. This shift in care offers opportunities for scaling up innovative and feasible options for the prevention and treatment of AUDs, which are major risk factors for several non-communicable diseases (NCDs).

Table 3 summarises the main characteristics of the studies reported in this review.

**Table 3:** Key characteristics of studies reported in this review

Author	Year	Country	Setting	Sample	N	Study design	Key findings
*Latin America*							
Carneiro	2018	Brazil	Online survey with completers of a distance learning course	Health professionals or social workers, who had completed a 120-h distance learning course on alcohol and drugs, screening and BI 83% were women from the South and Southeast (68%) regions Patients with ASSIST scores higher than 11 for alcohol or 4 for other drugs and aged 18 or above	2420 complete a online survey 25 of those implemented screening and BI 79 patients followed 3 months after receiving BI	Online survey with course attenders and follow-up of patients receiving SBI	Most of course completers used SBI in their work and felt very motivated to do it Patients receiving SBI shower lower alcohol and cocaine/crack scores in ASSIST 3 months after follow-up
Conde	2018	Argentina	Public secondary schools	Adolescents Age: M = 15.14, SD = 1.46 (Screening only: M:15.2; Screening and evaluation: M:15.2; Screening, evaluation and intervention: M:15) 81% Male (Screening only: 75%; Screening and evaluation: 80%; Screening, evaluation and intervention: 90%) Abstainers: 9% (Screening only: 8%; Screening and evaluation: 8%; Screening, evaluation and intervention: 12%)	167 (150 at follow-up)	RCT (two control groups (screening, screening and evaluation) and one experimental group (screening, assessment and intervention)	The intervention effectively reduced alcohol consumption and related problems in about one out of seven adolescents, with a minimal investment in training and implementation. However, we did not find significant differences in alcohol-related problems among the groups, which decreased under all conditions
Martínez-Martínez	2018	Mexico	350 Primary Care Units	Health professionals	756	Cross-sectional	Main barrier were amount of time taken to conduct an evaluation of the problem, that users do not complete the tasks assigned, their low educational, the user’s difficulties in going to the centre
Poblete	2017	Chile	9 primary care centres (n = 520), eight emergency rooms (n = 195) and five police stations (n = 91)	Non-treatment-seekers ASSIST scores higher than 11 for alcohol or 4 for other drugs 79% Male (Intervention); 78% Male (Control) Age: Mean 28.6 (SD: 7.8) Intervention; Mean 29.7 (SD: 8.3) (Control)	806 (400 Intervention, 406 Control)	Open-label parallel-group trial	No difference between the two groups for the ASSIST, alcohol, cannabis or cocaine
Reyes-Rodríguez	2017	Colombia	Secondary schools	Adolescents (11–18 years) 52% Females 44% last month alcohol use prevalence	3159	Longitudinal (follow-up conducted between 1 and 7 months after first session)	Participants of the preventive program brief intervention based on Motivational Interviewing showed a reduction of the frequency and quantity of alcohol consumption
Andrade	2016	Brazil	6 weeks web-based intervention to reduce alcohol use and related problems	Mean age 40 years 53.6% male 80.3% employed 68.6% college degree	929 accessed the intervention 94 with 6-weeks follow-up data	Longitudinal	Heavy users reduced their alcohol consumption by 30–50% after baseline Dependent alcohol users were more adherent to the intervention than Harmful users
Hoffman	2016	Peru	People living with HIV/AIDS	Tertiary hospital professionals attending people living with HIV/AIDS	Two focus groups: N = 51 Follow-up interviews after 6-months: N = 6	Qualitative interviews and focus groups	Main barriers to BI implementation were: (1) the unknown extent of substance use within PLWHA, (2) space and time limitations hinder completion of brief interventions during routine visits, and (3) insufficient number of services to refer patients to substance use treatment appropriate for HIV patients
Martínez Martínez	2016	Mexico	Workers from institutions providing BI	Purposive sample of key informants with experience with BI 28 to 57 years (Mean: 40.6, SD = 8.72) All Psychologists with 2–10 years of health and clinical experience	16	Qualitative interviews	Main barriers programs implementation were bureaucratic procedures and institutional policies, lack of knowledge of the theoretical bases of the program, and the diversity of users demanding the service
Moretti-Pires	2011	Brazil	Primary health care	136 health care professionals (9 doctors, 7 nurses, 120 health community agents) trained to use screening and BI	136 health care professionals 667 screened patients	Mixed methods (focus groups and epidemiological data)	25% of patients had a AUDIT score higher than 8 Main challenges to implement BI were the predominance of the biomedical approach, lack of continuity due to high professional rotation levels related to political reasons, difficulties to stabilize policies in places with limited access
Natera Rey	2011	Mexico	Community health centres	Alcohol user relatives 18–65 years Women only Small communities (340 habitants)	60	Quasi-experimental	The group that received the intervention showed a significant reduction in physical and psychological symptoms and depression
Martínez Martínez	2010	Mexico	Urban and rural adolescents attending high school	Adolescents (14–18 years) Binge drinkers in last 6 months, reporting at least one alcohol-related problem, and no dependence diagnosis	58	RCT (three groups: brief intervention, brief counselling and control group)	Both interventions groups showed reductions on alcohol use compared to control
Ronzani	2009	Brazil	Primary Care Units from two municipalities	Primary healthcare professionals and managers from three cities Age: Municipality A was M: 37 years; in the other two M: 30 years Sex: Municipality A 92.5% were female; in the other two 71.2% were females	113	Mixed methods	Managers engagement and healthcare professionals’ integration were associated with greater effectiveness in implementing alcohol prevention strategies
Martínez Martínez	2008a	Mexico	High school/College	Adolescents (14–18 years), Binge drinkers in last 6 months, reporting at least one alcohol-related problem, and no dependence diagnosis	40	RCT	BI group showed lower alcohol use compared to a control group 3- and 6-months post-treatment
Martínez Martínez	2008b	Mexico	High school	Adolescents (14–18 years) with alcohol/drug abuse 76% Male Age: M:16, SD:1.8 17 alcohol users and 8 cannabis users	25	Longitudinal (single group with 1, 3- and 6-months follow-up)	Results showed a reduction on alcohol and cannabis use at follow-up
Ronzani	2005	Brazil	Primary health care	Managers and primary health care professionals trained to use screening and BI	45 (5 managers; 40 health care professionals)	Qualitative interviews	Participants reported difficulties in routinely implementing BI; Health care professionals limited BI use to alcohol-dependent patients and demonstrate lack of motivation for preventive work
De Micheli	2004	Brazil	Outpatient treatment centre	Adolescents (10–19 years): COUM = M:15,5 (SD:2); CONUM = M:13 (SD:1,5); BI = M:15 (SD:1,5); PO = M:13,5 (SD:2) 50.5% Male Attenders of a outpatient care unit	99	RCT (four groups: a control group of users in the last month (COUM), a control group of non-users in the last month (CONUM), a Brief Intervention group (BI -in case they were regular users) and a Preventive Orientation group (PO—in case they were non users in the last month)	A single BI session with drug use showed a reduction on cannabis, alcohol and tobacco consumption after 6 months CONUM group showed at 6-month follow-up a significant increase in cannabis, alcohol and tobacco consumption, as well as in the intensity of related-problems
*India*							
Jhanjee	2017	India	Community	Female only sample Mean age: 43 (13) Illiterate (61%), homemakers (69%), nuclear family (60%), married (79.0%)	100	Pilot randomised controlled trial	BI group were two times more likely to stop tobacco use compared to control group (simple advice)
Nadkarni	2017	India	Primary care	Male only sample Mean age: 42.3 (11.8) treatment group and 41.7 (10.9) control group Married (78%) (treatment group) vs 154 (81%) (control group), employed: 163 (87%) (treatment group) vs 164 (87%) (control group), and completed at least primary education 147 (78%) (treatment group) vs 160 (85%) (control group)	377	Randomised controlled trial	Intervention was associated with short-term (over 3 months) and sustained effects (over 12 months) on drinking outcomes, including higher remission and 14-day abstinence rates
Humeniuk	2011	India	Community health centres	Male only sample Mean age: 31.4 (9.3) Average years of education: 9.5 (SD = 5.2) Employed (94%) Married (34%)	731 (total sample) 177 participants from India	Randomised controlled trial	BI group had significantly lower Alcohol, Smoking and Substance Involvement Screening Test (ASSIST) total scores for illicit substance involvement at follow-up compared with the control participants (wait-list control), with stronger effects on cannabis and opioid use scores at the India site
Pal	2007	India	Community	Male only sample Mean age: 29.7 years (9.89) Married: (67.7%)	90	Non-randomised controlled trial	Decrease in severity of dependence as measured by alcohol use in the last 30 days, composite alcohol severity index scores and improvement in physical and psychological quality of life, in those in the treatment group (BI) versus those in the control group (simple advice)

## Discussion

Although there have been some efforts to test and/or implement BIs in LMICs, as described above, much more needs to be done, specially taking into account that BIs are part of the five high-impact strategies to reduce alcohol problems included in the SAFER (Strengthen restrictions on availability, Advance and enforce drink driving counter measures, Facilitate access to screening, BIs and treatment, Enforce bans or comprehensive restrictions on alcohol advertising, sponsorship, and promotion, Raise prices of alcohol) initiative promoted by the World Health Organization (WHO). This is particularly important as not much is known about contextual influences, such as cross-cultural variability, and health system idiosyncrasies, or indeed how existing evidence, primarily from HICs, may generalise to other healthcare settings [72, 73].

Although there are examples of implementation of BI programmes, including those from Latin America described above, most efforts to implement BIs at scale have been largely unsuccessful [74]. There are several reasons for this, including conflicting priorities between a public health and prevention approach versus a clinical approach in which general practitioners may be more concerned with identifying and dealing with patients’ existing problems [75].

The evidence gap for AUDs in India needs urgent attention, with future work needing to focus on examining implementation and scale up concerns regarding BIs, and how systems can be better planned to integrate care for alcohol-related problems. First, a move away from the focus on tertiary-care for addictions is necessary, as it is not only expensive, but also has limited availability and access. Second, with most research in India being focused on BIs for tobacco use [76–78], expansion to other areas of addictions research is crucial. Third, there needs to be a move towards adoption and scaling up of contextually appropriate and evidence-based interventions. For instance, several studies have demonstrated the validity and utility of brief screening instruments (e.g. the Alcohol Use Disorders Identification Test) [79], and effectiveness of lay-counsellor delivered and primary-care based models for treatment of harmful drinking [67, 68]. BIs can play a key role in addressing the burden, harms and societal costs associated with AUDs in India. However, there is need for developing and evaluating new contextually relevant evidence in India, and also testing the implementation of evidence based BIs in this specific cultural context.

Hence, much more needs to be done in terms of research on BIs, both effectiveness and implementation, in LMICs given the uncertainties about their effects. However, the pace of such research in LMICs has been disappointing, despite clear evidence suggesting a growing burden of AUDs and the magnitude of unmet needs of people with such disorders. The first step towards overcoming this research gap is leadership and championing of these efforts, not just by addictions researchers and specialists, but by generalist clinicians and health care system managers within the larger NCD arena. In this context, examples of some promising initiatives include the screening and brief intervention programme based on the Alcohol, Smoking and Substance Involvement Screening Test (ASSIST) to help decrease substance misuse in primary care in Thailand [80], and the SCALA program in Colombia, Mexico and Peru.

Innovative strategies need to be deployed to overcome supply side barriers related to specialist workforce shortages in LMICs. There is a growing evidence on the effectiveness of non-specialist health workers, including lay counsellors, in delivering frontline psychological interventions for a range of disorders including AUDs in LMICs [81]. The potential of such a non-specialist cadre of workers needs to be appropriately leveraged through training, monitoring and hand-holding of such personnel. Additionally, there is growing evidence about the utility of technology based interventions for a range of substance use problems [82]. This is true for LMICs as well, where technology is increasingly providing new possibilities for delivering a range of interventions [83]. In many LMICs there has been a technological leap with a ‘mobile-first’-based approach to communications, and people are more likely to have access to a mobile phone than to have clean water, or a source of electricity [76]. As the market penetration of low-cost mobile devices increases in such countries it provides another potentially transformative opportunity for increasing access to BIs. Finally, in addition to increasing access, it is also crucial to reduce demand side barriers by reframing AUDs as a preventable health risk, especially in LMICs where they are often seen as a character flaw or a moral issue.

One might question the utility of examining the barriers to implementation of BIs in LMICs when these have not been implemented in regular practice anywhere else in the world. Presently there is evidence for a modest effect of BIs in efficacy studies in HICs, while their implementation in regular health care remains elusive. The reasons for this are many, the most important being that the concept of screening and brief intervention comes from public health, but hopes to be applied in clinical practice. The major problems with this are well known: practitioners do not like general screening, because they do not feel they have time for alcohol discussions and furthermore they lack confidence in their ability to help problem drinkers. The barriers to implementation discussed in this paper largely apply to high income countries (HICs) as well: lack of time, lack of skills, lack of perceived relevance, etc. These are observations that call for major rethinking of how brief interventions should be conducted—in LMICs as well as in HICs. Table 4 summarises the key findings of our paper, its implications, and the way forward.

**Table 4:** Summary points

Summary points
Research in context: The harmful use of alcohol is projected to increase globally, and particularly substantially in LMICs, indicating the need for comprehensive interventions to address the burgeoning burden of alcohol-related harms. An intervention with demonstrated effectiveness and cost-effectiveness evidence from diverse settings is the screening and BI approach for alcohol-related problems. Despite positive evidence favouring the use of BIs, a major challenge in the field of addictions has been the effective deployment and evaluation of this approach in routine practice
Added value of this study: Our paper aims to synthesise the state of the evidence for the implementation of BIs for alcohol use disorders, particularly hazardous and harmful drinking. We focus on low-resource settings because of challenges related to the uptake, dissemination and implementation of evidence-based BIs in these settings and provide potential solutions with specific examples from two low-resource contexts
Implications of existing evidence: To the best of our knowledge, there have been very few efforts to examine bottlenecks and opportunities in BI research in LMIC contexts. There is promise in potential solutions for supply-side barriers in care provision, including the use of the task-sharing model, digital technology-based delivery, remote capacity building efforts, etc. There is also a need for further work to unpack how BIs can be implemented and optimized in low-resource settings

We hope that our paper, a collaborative exercise between early-career and senior researchers from LMIC and HICs, in the context of the International Network on Brief Interventions for Alcohol and Other Drugs (www.inebria.net), stimulates discussions among policy-makers, researchers, and practitioners in LMICs. Increasing access to evidence based care for AUDs in LMICs would need a concerted effort from all these stakeholders, especially with regard to the systemic and philosophical changes to research and implementation strategies underlying prevention efforts.

## Conclusion

In conclusion, the evidence on implementation of BIs in LMICs is limited. Despite the global evidence of efficacy, there are several systemic barriers to implementation and scaling up BIs in such countries. Our paper highlights these barriers and also makes the case for the implementation and rigorous evaluation of innovative strategies to enable the scaling up of BIs despite the limited resources in LMICs.

## Data Availability

Data sharing not applicable to this article as no datasets were generated or analysed during the current study.

## Abbreviations

AUD: Alcohol use disorders
BI: Brief interventions
ASSIST: Alcohol, Smoking and Substance Involvement Screening Test
HIC: High income countries
LMIC: Low- and middle-income countries
MI: Motivational interviewing
NCD: Non communicable diseases
WHO: World Health Organization
